# Association of Untargeted Urinary Metabolomics and Lung Cancer Risk Among Never-Smoking Women in China

**DOI:** 10.1001/jamanetworkopen.2019.11970

**Published:** 2019-09-20

**Authors:** Wei Jie Seow, Xiao-Ou Shu, Jeremy K. Nicholson, Elaine Holmes, Douglas I. Walker, Wei Hu, Qiuyin Cai, Yu-Tang Gao, Yong-Bing Xiang, Steven C. Moore, Bryan A. Bassig, Jason Y. Y. Wong, Jinming Zhang, Bu-Tian Ji, Claire L. Boulangé, Manuja Kaluarachchi, Anisha Wijeyesekera, Wei Zheng, Paul Elliott, Nathaniel Rothman, Qing Lan

**Affiliations:** 1 Saw Swee Hock School of Public Health, National University of Singapore and National University Health System, Singapore; 2 Department of Medicine, Yong Loo Lin School of Medicine, National University of Singapore and National University Health System, Singapore; 3 Division of Cancer Epidemiology and Genetics, National Cancer Institute, National Institutes of Health, Rockville, Maryland; 4 Division of Epidemiology, Department of Medicine, Vanderbilt University Medical Center and Vanderbilt-Ingram Cancer Center, Nashville, Tennessee; 5 Biomolecular Medicine, Division of Computational and Systems Medicine, Medical Research Council–National Institute for Health Research National Phenome Centre, Imperial College London, United Kingdom; 6 Medical Research Council–PHE Centre for Environment and Health, Department of Surgery and Cancer, Imperial College London, United Kingdom; 7 Health Futures Institute, Murdoch University, Murdoch, Western Australia, Australia; 8 Department of Environmental Medicine and Public Health, Icahn School of Medicine at Mount Sinai, New York, New York; 9 Department of Epidemiology, Shanghai Cancer Institute, Shanghai, China; 10 State Key Laboratory of Oncogene and Related Genes, Renji Hospital, Shanghai Jiaotong University School of Medicine, Shanghai, China; 11 MRC-PHE Centre for Environment and Health, School of Public Health, Department of Epidemiology and Biostatistics, Imperial College London, United Kingdom; 12 National Institute for Health Research, Imperial College Biomedical Research Centre, London, United Kingdom; 13 Health Data Research UK London at Imperial College London, United Kingdom

## Abstract

**Importance:**

Chinese women have the highest rate of lung cancer among female never-smokers in the world, and the etiology is poorly understood.

**Objective:**

To assess the association between metabolomics and lung cancer risk among never-smoking women.

**Design, Setting, and Participants:**

This nested case-control study included 275 never-smoking female patients with lung cancer and 289 never-smoking cancer-free control participants from the prospective Shanghai Women’s Health Study recruited from December 28, 1996, to May 23, 2000. Validated food frequency questionnaires were used for the collection of dietary information. Metabolomic analysis was conducted from November 13, 2015, to January 6, 2016. Data analysis was conducted from January 6, 2016, to November 29, 2018.

**Exposures:**

Untargeted ultra-high-performance liquid chromatography–tandem mass spectrometry and nuclear magnetic resonance metabolomic profiles were characterized using prediagnosis urine samples. A total of 39 416 metabolites were measured.

**Main Outcomes and Measures:**

Incident lung cancer.

**Results:**

Among the 564 women, those who developed lung cancer (275 participants; median [interquartile range] age, 61.0 [52-65] years) and those who did not develop lung cancer (289 participants; median [interquartile range] age, 62.0 [53-66] years) at follow-up (median [interquartile range] follow-up, 10.9 [9.0-11.7] years) were similar in terms of their secondhand smoke exposure, history of respiratory diseases, and body mass index. A peak metabolite, identified as 5-methyl-2-furoic acid, was significantly associated with lower lung cancer risk (odds ratio, 0.57 [95% CI, 0.46-0.72]; *P* < .001; false discovery rate = 0.039). Furthermore, this peak was weakly correlated with self-reported dietary soy intake (ρ = 0.21; *P* < .001). Increasing tertiles of this metabolite were associated with lower lung cancer risk (in comparison with first tertile, odds ratio for second tertile, 0.52 [95% CI, 0.34-0.80]; and odds ratio for third tertile, 0.46 [95% CI, 0.30-0.70]), and the association was consistent across different histological subtypes and follow-up times. Additionally, metabolic pathway analysis found several systemic biological alterations that were associated with lung cancer risk, including 1-carbon metabolism, nucleotide metabolism, oxidative stress, and inflammation.

**Conclusions and Relevance:**

This prospective study of the untargeted urinary metabolome and lung cancer among never-smoking women in China provides support for the hypothesis that soy-based metabolites are associated with lower lung cancer risk in never-smoking women and suggests that biological processes linked to air pollution may be associated with higher lung cancer risk in this population.

## Key Points

**Question:**

What is the association of metabolomic biomarkers with lung cancer in women who have never smoked?

**Findings:**

In this case-control study of 275 never-smoking women with lung cancer and 289 never-smoking cancer-free women in China, the metabolite 5-methyl-2-furoic acid was correlated with dietary soy consumption and pathways including 1-carbon metabolism, oxidative stress, nucleotide metabolism, and inflammation and was significantly associated with lower lung cancer risk.

**Meaning:**

These findings suggest that certain metabolites and pathways are associated with lower lung cancer risk in never-smoking women and biological processes linked to air pollution may be associated with higher lung cancer risk in this population.

## Teaser

This case-control study investigates the association between urinary metabolomic biomarkers and lung cancer risk among never-smoking women in China.

## Introduction

Lung cancer is the leading cause of cancer mortality among men and women, accounting for 27% of all cancer deaths worldwide and nearly 158 040 deaths in the United States in 2015.^1^ Although most cases of lung cancer are caused by active tobacco smoking, approximately 25% occur in never-smokers.^2^ Asian individuals (particularly Chinese women) have a higher rate of lung cancer among never-smokers compared with individuals of European descent^3^; however, the etiology and risk factors of this malignant neoplasm independent of smoking are poorly understood.^4^ Therefore, it is imperative to understand the etiology of lung cancer and identify new biomarker targets or metabolomic profiles as potential diagnostic and screening tools for lung cancer.

Altered tumor metabolism, a distinctive hallmark of many cancers, is required to acquire sufficient nutrients to maintain deregulated and unlimited proliferation of the cancer cells.^5^ An agnostic approach using untargeted metabolic profiling allows more comprehensive coverage of downstream biological responses to environmental exposures, and can provide insight into underlying molecular disease mechanisms.^6^ Metabolomic techniques have been used to identify metabolites in various cancers.^7,8,9,10,11^ However, most of these metabolomic studies of cancer were conducted using case-control study designs that compared the metabolite levels between individuals diagnosed with cancer and healthy controls, which could be prone to reverse causation bias. Targeted prospective studies have found some urinary metabolites to be associated with higher lung cancer risk, including creatine riboside,^12^ N-acetylneuraminic acid,^12^ and 7-methylguanine.^13^ Both studies were conducted in populations of European descent and had small numbers of never-smokers.

The Shanghai Women’s Health Study (SWHS) is a population-based prospective cohort of 73 363 female participants who were predominantly never-smokers. This study provides a unique opportunity to prospectively evaluate the role of the metabolome in lung cancer development. To our knowledge, this is the first prospective study to investigate prediagnostic metabolic biomarkers and pathway alterations in urine samples using an untargeted approach among never-smoking women, which may provide novel insights into the etiology and mechanisms associated with lung cancer development in never-smokers.

## Methods

### Study Population

From December 28, 1996, to May 23, 2000, the SWHS recruited 73 363 Chinese women aged 40 to 70 years who were primarily never-smokers. In this nested case-control study, we performed metabolomic profiling of all 275 female patients with incident lung cancer and 289 female healthy controls who were individually matched on date of birth (±2 years) and date of spot urine sample collection (±3 months). We followed the Strengthening the Reporting of Observational Studies in Epidemiology (STROBE) reporting guideline. Information on demographic characteristics, lifestyle, diet, and medical history was obtained using a detailed survey administered in person at baseline.^14^ A comprehensive and validated food frequency questionnaire was used to assess dietary intake during the year prior to the interview. Urine samples were collected into a sterilized cup containing 125 mg of ascorbic acid to prevent oxidation of labile metabolites. After collection, the samples were kept at approximately 0°C to –4°C and were processed within 6 hours for long-term storage at −70°C.^14^ All study participants provided written informed consent prior to participation, and the study protocols were approved by institutional review boards of all participating institutes and the National Cancer Institute.

### Follow-up and Lung Cancer Identification

The participants were followed up with a combination of in-person surveys and periodic linkage to cancer and vital statistics registries.^14^ Briefly, cohort members were followed up for diagnosis of cancer by in-person surveys every 2 to 3 years and annual record linkage with databases from the population-based Shanghai Cancer Registry, Shanghai Vital Statistics Registry, and Shanghai Resident Registry. All possible cancer diagnoses were verified through home visits and a review of medical records by a panel of oncologists. Lung cancer was defined as code 162 under the *International Classification of Diseases, Ninth Revision*. All female patients with lung cancer were included as soon as they were diagnosed after the baseline recruitment through December 31, 2009. Lung cancer diagnosed before the recruitment was excluded. Information on histological subtypes of lung cancer was extracted from medical records. Histological groups of lung cancer were classified according to the morphology code of *International Classification of Diseases for Oncology, Second Edition* (8140/3).^15^

### Urinary Metabolomics

All urine samples (patient and pooled quality control samples) were analyzed in an untargeted fashion by ultra-high-performance liquid chromatography–tandem mass spectrometry (UPLC-MS) and 600-MHz hydrogen 1 nuclear magnetic resonance (NMR) spectroscopy at Imperial College London, United Kingdom, acquired between November 13, 2015, and January 6, 2016. The spectral data for the pooled quality control sample was collected after every 5 patient samples. The measurements were carried out according to established protocols used for metabolic phenotyping of biofluids.^16,17^ For the UPLC-MS measurements, metabolic profiling was performed on an ACQUITY UPLC System (Waters Corp) coupled to a Xevo Triple Quadrupole Mass Spectrometer (Waters Corp) following a standard set of protocols.^18^ Mass spectrometry was performed using electrospray in both positive and negative ionization (electrospray ionization [ESI]+ and ESI−) modes under conditions described previously.^19^ This UPLC-MS method allows for a high-resolution, accurate, and simultaneous detection of thousands of peaks that correspond to individual ions with a unique mass to charge (m/z) ratio and retention time.^20^

Each UPLC-MS data set was preprocessed using Progenisis (Non Linear Dynamics) to convert the 3-dimensional UPLC-MS raw data into time-aligned detected features of retention time, m/z ratio, and intensity (peak area). The ion intensities for each peak detected were then normalized, within each sample, to the median peak intensity in that sample. The metabolic profiling data were preprocessed using the freely available XCMS software (Scripps Research Institute) to detect and identify peaks, align retention time, and perform normalization of the raw mass spectrometry data before subsequent statistical analysis.^19,21^ The resulting features were filtered to remove features with a coefficient of variation greater than 30% across the quality control samples. The NMR data were imported into MATLAB R2012b (Natick) using the Metaspectra program script, using a resolution of 0.00055. The data were then aligned using recursive segmentwise peak alignment^22^ and normalized by probabilistic quotient to account for and exclude systematic sources of bias within samples not due to biological processes or the environment. Statistical recoupling of variables^23^ was then used for peak detection. The resulting tables were imported to SIMCA-P+ software version 13.0.2 (Umetrics) to perform multivariate statistical analysis. In total, there were 20 555 UPLC-MS peaks in positive mode, 18 475 in negative mode, and 386 NMR peaks.

### Statistical Analysis 

A small constant (1 × 10^−9^) was added to all metabolite levels, which were then natural log–transformed to approximate normal distributions and subsequently analyzed as both continuous and categorical variables (based on tertiles among controls). Principal component analysis, partial least-squares–discriminant analysis, and orthogonal projection on latent structures–discriminant analysis (OPLS-DA) were performed on all data to check for batch effects and clustering of samples.

The characteristics of patients with lung cancer and control individuals were compared using the Wilcoxon rank sum test for continuous variables and Fisher exact test for categorical variables. Analyses were restricted to lifetime never-smokers (275 female patients with lung cancer and 289 controls). Separate unconditional logistic regression models were used to estimate the odds ratios (ORs) and 95% confidence intervals for the associations between each metabolite and lung cancer risk. Tests of trend were calculated by treating the level of each metabolite as a continuous variable. We also stratified the analysis by follow-up time (<9 years [the median duration of follow-up] or ≥9 years) and major lung cancer subtypes (adenocarcinoma, nonadenocarcinoma). Because dietary intake has previously been reported to be associated with altered metabolites in urine samples, using a multivariable linear regression model, we evaluated the associations between the most significant metabolite and prediagnosis dietary factors among female patients with lung cancer and controls. In addition, because soy food intake has been previously found to be associated with risk of lung cancer in the SWHS,^24^ we further looked at grouped soy intake, which included 11 soy food items that are commonly consumed in Shanghai, including soy milk, tofu, fried tofu, dried or pressed tofu, fresh green soy beans, dry soy beans, soy sprouts, and other soy products. In addition, mediation analysis was used to estimate the proportion of the mediated effect via the significant metabolites on the association between soy consumption and lung cancer risk.^25^ Missing data were excluded from all analyses. All models were adjusted for age (continuous), body mass index, history of respiratory diseases (categorical; ever or never) and environmental tobacco smoke (categorical; ever or never). Correlations among significant metabolites, as well as between significant metabolites and 17 dietary factors, were evaluated using Spearman correlation coefficients.

All statistical analyses were conducted using R statistical software version 3.2.2 (R Project for Statistical Computing) and SAS statistical software version 4.3 (SAS Institute). All tests with a false discovery rate (FDR) of less than 0.10 were considered statistically significant. All *P* values were reported based on 2-sided tests and *P* < .05 was considered statistically significant. We calculated FDRs using the Benjamini-Hochberg method to account for multiple comparisons.^26^

### Metabolite Identification

The metabolic phenotypes of female patients with lung cancer and controls were further evaluated using pathway enrichment to identify biological processes associated with lung cancer diagnosis. In this analysis, we tested all metabolic features for their association with lung cancer diagnosis. Pathway enrichment analysis was completed using all metabolic features with *P* < .05. This threshold minimizes type II error (false-negatives) at the increased risk of type I error (false-positives). The pathway enrichment approach uses a random sampling framework to identify a null distribution of metabolites across a metabolic network; comparison of the null distribution with annotated metabolites from signals with *P* < .05 removes false-positives, which would be randomly distributed across the metabolic map, and allows isolation of biological effects. The pathway enrichment was completed separately for positive- and negative-mode data using the mummichog analysis approach^27^; pathways with *P* < .05 were selected for further characterization. Metabolites were identified and annotated based on accurate mass (for UPLC-MS) and by using in-house and available online databases such as the Human Metabolome Database and METLIN.

## Results

Among the 564 women, those who developed lung cancer (275 participants; median [interquartile range {IQR}] age, 61.0 [52-65] years) and those who did not develop lung cancer (289 participants; median [IQR] age, 62.0 [53-66] years) at follow-up were similar in terms of secondhand smoke exposure, history of respiratory diseases, and body mass index (Table 1). The median (IQR) time from the baseline questionnaire to lung cancer diagnosis was 9.26 (6.69-11.05) years.

**Table 1.** Characteristics of the Nested Case-Control Study of Never-Smoking Women in the Shanghai Women’s Health Study

Characteristic	Lung Cancer (n = 275)	No Lung Cancer (n = 289)	*P* Value^a^
Environmental tobacco smoke, No. (%)			
Never	70 (25.5)	71 (24.6)	.86
Ever	187 (68)	200 (69.2)	
Missing	18 (6.5)	18 (6.2)	
History of respiratory diseases, No. (%)			
Yes	28 (10.2)	22 (7.6)	.36
No	247 (89.8)	267 (92.4)	
Age at baseline, median (IQR), y	61.0 (52-65)	62.0 (53-66)	.42
BMI, median (IQR)	24.3 (22.4-26.6)	24.3 (22.0-26.9)	.84
Time to diagnosis, median (IQR), y	9.26 (6.69-11.1)	NA	NA
Histological subtypes, No. (%)			
Adenocarcinoma	135 (49.1)	NA	NA
Squamous cell carcinoma	9 (3.3)	NA	
Other histology subtypes	19 (6.9)	NA	
Unclassified	104 (37.8)	NA	
No *ICD-O-2* code	8 (2.9)	NA	

Abbreviations: BMI, body mass index (calculated as weight in kilograms divided by height in meters squared); *ICD-O-2*, *International Classification of Diseases for Oncology, Second Revision*; IQR, interquartile range; NA, not applicable.

^a^ *P* values were obtained from Wilcoxon signed-rank test for continuous variables and Fisher exact test for categorical variables.

Using an FDR cutoff of 10% to adjust for multiple comparisons, 3 urinary metabolites by UPLC-MS were significantly associated with lower lung cancer risk (Table 2). The statistically significant metabolites were pos_2.61_127.0382m/z (OR, 0.57 [95% CI, 0.46-0.72]; *P* < .001; FDR = 0.039), neg_2.60_369.0408m/z (OR, 0.97 [95% CI, 0.96-0.98]; *P* < .001; FDR = 0.039), and pos_2.61_184.0325n (OR, 0.55 [95% CI, 0.43-0.71]; *P* < .001; FDR = 0.065). The 3 significant metabolites were highly correlated with each other (Spearman correlations ranged from 0.67-0.89) (eTable 1 in the Supplement). In addition, results were comparable when stratified by follow-up time (<9 years of follow-up and ≥9 years of follow-up) after urine sample collection (Table 2). Similar results were also obtained after excluding 4 women who were diagnosed within 2 years after the baseline interview (data not shown). Furthermore, similar associations were observed among patients with lung adenocarcinoma (135 patients [49.1%]) and those with unclassified lung cancer (104 patients [37.8%]) (eTable 2 and eTable 3 in the Supplement). None of the NMR features were significantly associated with lung cancer risk (data not shown).

**Table 2.** Associations Between Urinary Metabolites and Lung Cancer Risk, Stratified by Follow-up Time

Metabolite^a^	OR (95% CI)^b^	*P* Value	FDR	Follow-up			
				<9 y^c^		≥9 y^d^	
				OR (95% CI)^b^	*P* Value	OR (95% CI)^b^	*P* Value
pos_2.61_127.0382m/z	0.57 (0.46-0.72)	<.001	0.039	0.51 (0.38-0.70)	<.001	0.62 (0.48-0.81)	<.001
neg_2.60_369.0408m/z	0.97 (0.96-0.98)	<.001	0.039	0.97 (0.95-0.98)	<.001	0.97 (0.96-0.99)	<.001
pos_2.61_184.0325n	0.55 (0.43-0.71)	<.001	0.065	0.49 (0.35-0.69)	<.001	0.60 (0.44-0.82)	.001

Abbreviations: FDR, false discovery rate; OR, odds ratio.

^a^ Metabolites were natural log–transformed.

^b^ All models were adjusted for age, body mass index, history of respiratory diseases, and secondhand smoke exposure.

^c^ Sample sizes were 131 with lung cancer and 289 without lung cancer.

^d^ Sample sizes were 144 with lung cancer and 289 without lung cancer.

One of the significant metabolites (pos_2.61_127.0382m/z) was identified as 5-methyl-2-furoic acid based on its fragmentation pattern by tandem mass spectrometry (eTable 4 in the Supplement). There was a significant monotonic dose-response association between 5-methyl-2-furoic acid and lung cancer risk when analyzed as tertiles (Table 3). Increasing tertiles of the metabolite level were significantly associated with lower lung cancer risk (in comparison with first tertile, OR for second tertile, 0.52 [95% CI, 0.34-0.80]; and OR for third tertile, 0.46 [95% CI, 0.30-0.70]; *P* for trend < .001). Similar trends were observed for the 2 other significant metabolites (eTable 5 in the Supplement).

**Table 3.** Associations Between 5-Methyl-2-Furoic Acid and Lung Cancer, by Tertiles and Stratified by Follow-up Time

Log Metabolite Tertiles	Patients With Lung Cancer, No.	Patients Without Lung Cancer, No.	OR (95% CI)^a^	*P* Value	*P* for Trend
First tertile, median = 10.5	139	96	1 [Reference]	NA	<.001
Second tertile, median = 11.3	71	95	0.52 (0.34-0.80)	.003	
Third tertile, median = 12.1	64	96	0.46 (0.30-0.70)	<.001	
<9-y follow-up					
First tertile, median = 10.5	69	96	1 [Reference]	NA	<.001
Second tertile, median = 11.3	34	96	0.51 (0.30-0.86)	.01	
Third tertile, median = 12.1	27	96	0.36 (0.20-0.63)	<.001	
≥9-y follow-up					
First tertile, median = 10.4	70	96	1 [Reference]	NA	.02
Second tertile, median = 11.2	37	96	0.53 (0.32-0.89)	.02	
Third tertile, median = 12.1	37	96	0.56 (0.34-0.93)	.03	

Abbreviations: OR, odds ratio; NA, not applicable.

^a^ All models were adjusted for age, body mass index, history of respiratory diseases, and secondhand smoke exposure.

Because dietary intake has been previously reported to be associated with altered metabolites in urine samples, we also assessed the correlation of each available dietary factor with the level of 5-methyl-2-furoic acid. Consumption of soy food, including soy milk, tofu, fried tofu, dried or pressed tofu, fresh green soy beans, dry soy beans, soy sprouts, and other soy products, in the past year had a weak positive correlation with levels of 5-methyl-2-furoic acid among controls (ρ = 0.21; *P* < .001) (eTable 6 in the Supplement). Using mediation analysis, we found that 25% of the association between soy consumption and higher lung cancer risk was significantly mediated via 5-methyl-2-furoic acid.

Regression analysis included 152 positive-mode and 186 negative-mode features associated with lung cancer risk at *P* < .05; only 4 features were present with Bonferroni correction less than .05. Pathway enrichment identified 40 metabolic pathways associated with lung cancer risk at *P* < .05, including 8 pathways meeting a Bonferroni-corrected *P* < .05 (Figure). Eight were detected in both modes and included amino acid metabolism, nucleotide metabolism, nitrogen catabolism, and B vitamin pathways. Metabolic alterations associated with lung cancer risk suggest systemic changes to key biological processes, including 1-carbon metabolism (vitamin B_9_ [folate] metabolism; glycine, serine, alanine, and threonine metabolism), oxidative stress pathways (ascorbate [vitamin C] and aldarate metabolism, methionine and cysteine metabolism, glutamate metabolism), nucleotide metabolism (purine metabolism), and inflammation (leukotriene metabolism).

**Figure.**
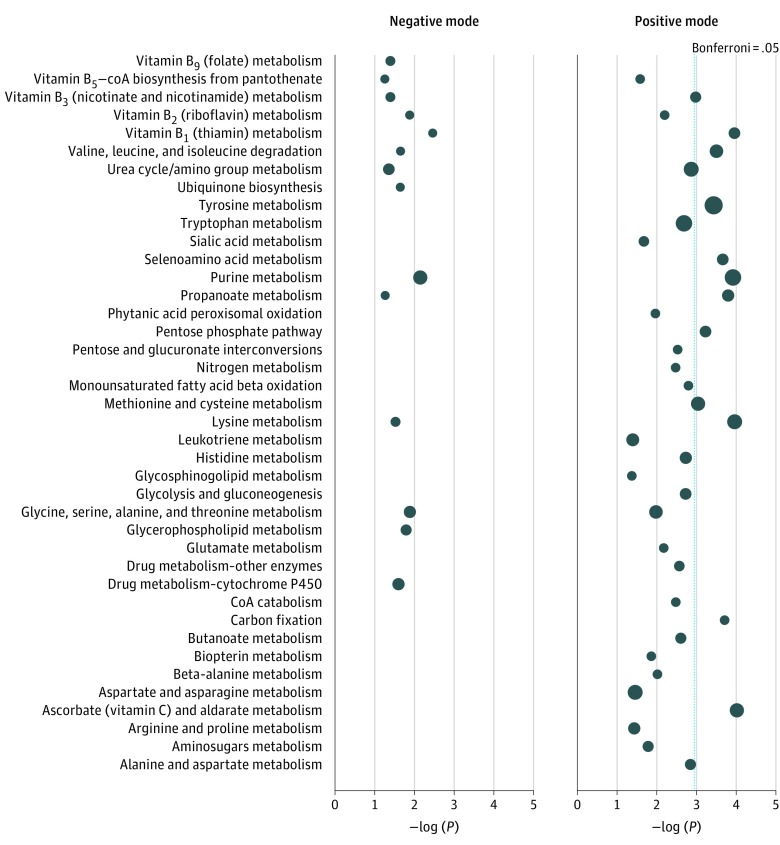
Metabolic Pathways Associated With Lung Cancer Risk Using Features With *P* < .05 Pathway enrichment was identified using the mummichog analysis approach and identified 40 metabolic pathways associated with lung cancer risk, including 8 pathways meeting a Bonferroni-corrected *P* < .05. Circle size is proportional to the number of metabolites associated with lung cancer diagnosis from each pathway. The placement of circles along the x-axis indicate the statistical significance of each pathway. The dotted line indicates the significance level using Bonferroni *P* = .05. CoA indicates coenzyme A.

## Discussion

To our knowledge, this is the first large-scale prospective study of untargeted de novo metabolomics in association with lung cancer among never-smoking Chinese women. We found 3 metabolites associated with a reduction of lung cancer risk after accounting for multiple comparisons. These metabolites were highly correlated with each other and may represent the same chemical family and/or source. One of these metabolites (pos_2.61_127.0382m/z) was identified as 5-methyl-2-furoic acid, which belongs to the family of furoic acid derivatives. It was weakly correlated with dietary soy intake in our study population, suggesting that this metabolite may be partially derived from soy-related dietary products. Metabolic alterations identified in the pathway enrichment analysis suggest lung cancer risk is associated with an increased oxidative state and depletion of systemic antioxidant pathways.

Furan fatty acids are related compounds that occur naturally in foods such as soybean oil^28^ and have also been shown to have anti-inflammatory and antioxidant properties in vivo.^29^ Methylated and furan-containing fatty acids have also been demonstrated to protect against reactive oxygen species–mediated damage in vivo by acting as scavengers of reactive oxygen species.^30^ Interestingly, we found higher levels of the furoic acid metabolite in urine to be associated with a lower risk of lung cancer. Another furoic acid derivative, 5-tetradecyl-oxy-2-furoic acid (TOFA), has been reported to reduce fatty acid synthesis by inhibiting acetyl coenzyme A carboxylase-α (ACCA), thereby increasing apoptosis rates and eradicating prostate cancer cells via the mitochondrial pathway.^31^ One study^32^ identified TOFA as a potent inhibitor of stearoyl coenzyme A desaturase 1 (SCD1), which is an enzyme in the fatty-acid synthesis pathway that depletes monounsaturated fatty acids and therefore restricts cancer cell proliferation. Furoic acid is derived from furans, which are commonly used as food additives, and preservatives, which are usually present in high levels in heat-treated foods such as soy sauce, baked beans, and brewed coffee.^33^

Dietary soy intake was previously shown to be associated with a 37% lower risk of lung cancer among a population of Chinese women in Shanghai, comparing the highest quintile with the lowest quintile.^24^ This is consistent with our findings in the same population of a significant protective association between 5-methyl furan-2-carboxylic acid and lung cancer risk, and a significant weak correlation between this metabolite and dietary soy intake. Soy foods contain high concentrations of isoflavones, a type of phytoestrogen, which possess antiestrogenic and antioxidative effects.^34^ It has been hypothesized that the bioactive compounds of dietary soy may contain antioxidant and anti-inflammatory properties that inhibit tumor growth and invasion and induce apoptosis.^35,36,37^

Evaluating pathway (rather than individual metabolite) changes associated with disease risk provides a systems biology approach that can be used to understand the metabolic effects of lung cancer pathobiology. In the present study, we identified alterations in pathways that suggest dysregulation of 1-carbon metabolism and related pathways, including folate, methionine and cysteine metabolism, purine metabolism, and glycine, serine, alanine, and threonine metabolism. One-carbon metabolism is critical in intermediary metabolism and supports multiple biological processes, including nucleotide metabolism, amino acid metabolism, and homeostasis (methionine and cysteine, serine, and glycine).^38^

Our study suggests that metabolic alterations in oxidative stress and inflammatory pathways, which could arise from multiple exogenous and endogenous exposures and biological processes, are associated with lung cancer risk in never-smoking women in China. It is, however, intriguing that these same pathways have also been linked to exposure to environmental pollutants that are suspected to be associated with higher lung cancer disease risk,^39,40^ including traffic-related air pollution.^41,42,43,44^ Taken together, the overlap in oxidative stress and inflammation metabolites suggest environmental exposures and lung cancer risk in this population may share similarities in metabolic alterations and provide an indirect link between the two. Additional studies are needed to evaluate these possible associations.

One major strength of this study is that the metabolites were measured in never-smoking women, thereby eliminating the possibility of confounding by active smoking, which has occurred in other studies. Other strengths of this study include the prospective design, in which the collection of urinary metabolomic biomarkers preceded lung cancer diagnosis, which provides exposure-outcome temporality, and the untargeted measurement of metabolites, which provides increased opportunities for making novel discoveries.

### Limitations

There were limitations to this study. One is that a notable proportion of the cohort with lung cancer (40.7%) had an unclassified histological subtype. However, analysis of unclassified lung cancer yielded results that were similar to analysis of all lung cancer and of lung adenocarcinoma only. The source of the dietary data was a food frequency questionnaire designed to assess dietary patterns in the past year when the baseline interview was conducted. The exact question asked in the questionnaire was, “Would you please tell me if you ate those foods and how much you ate in general over the past year?” Given that most metabolites have relatively short half-lives, the weak correlation between 5-methyl-2-furoic acid and dietary soy intake may have been stronger if we had questionnaire or diary data for the last few days before urine was collected. In addition, the urine samples were only collected once and we were unable to assess the temporal trends of metabolites over time.

## Conclusions

This nested case-control study found that a urinary metabolite, 5-methyl-2-furoic acid, was associated with decreased risk of lung cancer in a prospective cohort of never-smoking women. The identified significant metabolite (5-methyl-2-furoic acid) was weakly correlated with self-reported usual dietary intake of soy. Furthermore, our finding is consistent with recent epidemiological evidence showing that higher dietary soy consumption may be associated with decreased lung cancer risk among never-smokers, strengthening the biological relevance of the identified metabolite. In addition, metabolic pathways associated with lung cancer risk suggest systemic biological alterations were present, and a number of pathways identified in the present study have been linked to results of previous metabolomic studies of populations exposed to air pollution, a suspected risk factor for lung cancer. Additional studies are warranted to replicate and further characterize the associations reported in our study, which provide additional clues about the exposures and biological processes that may contribute to the risk of lung cancer in never-smoking populations in Asia.

## Notes

Supplement. **eTable 1.** Spearman Correlations Between Significant Metabolites (<10% FDR) in Controls Only, Cases Only and Overall Study Population **eTable 2.** Associations Between Urinary Metabolites and Lung Cancer Risk, Stratified by Lung Cancer Subtypes **eTable 3.** Associations Between the Most Significant Metabolite (5-Methyl-2-Furoic Acid) and Lung Cancer in Tertiles, Stratified by Lung Cancer Subtypes **eTable 4.** Identification of the Top Three Metabolites **eTable 5.** Associations Between neg_2.60_369.0408mz, pos_2.61_184.0325n and Lung Cancer, by Tertiles **eTable 6.** Univariate Linear Regression and Correlation Between the Most Significant Metabolite (5-Methyl-2-Furoic Acid) and Diet Variables Among Controls
