# Supplementary material for: Association of Untargeted Urinary Metabolomics and Lung Cancer Risk Among Never-Smoking Women in China
Source: JAMA Netw Open. 2019 Sep 20;2(9):e1911970. doi: 10.1001/jamanetworkopen.2019.11970 (PMC6755532; doi:10.1001/jamanetworkopen.2019.11970)
Supplement: Supplement. — eTable 1. Spearman Correlations Between Significant Metabolites (<10% FDR) in Controls Only, Cases Only and Overall Study Population eTable 2. Associations Between Urinary Metabolites and Lung Cancer Risk, Stratified by Lung Cancer Subtypes eTable 3. Associations Between the Most Significant Metabolite (5-Methyl-2-Furoic Acid) and Lung Cancer in Tertiles, Stratified by Lung Cancer Subtypes eTable 4. Identification of the Top Three Metabolites eTable 5. Associations Between neg_2.60_369.0408mz, pos_2.61_184.0325n and Lung Cancer, by Tertiles eTable 6. Univariate Linear Regression and Correlation Between the Most Significant Metabolite (5-Methyl-2-Furoic Acid) and Diet Variables Among Controls [file jamanetwopen-2-e1911970-s001.pdf]

Supplementary Online Content

Seow WJ, Shu X-O, Nicholson JK, et al. Association of untargeted urinary metabolomics and lung cancer risk among never-smoking women in China. *JAMA Netw Open*. 2019;2(9):e1911970. doi:10.1001/jamanetworkopen.2019.11970

eTable 1. Spearman Correlations Between Significant Metabolites (<10% FDR) in Controls Only, Cases Only and Overall Study Population
eTable 2. Associations Between Urinary Metabolites and Lung Cancer Risk, Stratified by Lung Cancer Subtypes
eTable 3. Associations Between the Most Significant Metabolite (5-Methyl-2-Furoic Acid) and Lung Cancer in Tertiles, Stratified by Lung Cancer Subtypes
eTable 4. Identification of the Top Three Metabolites
eTable 5. Associations Between neg_2.60_369.0408mz, pos_2.61_184.0325n and Lung Cancer, by Tertiles
eTable 6. Univariate Linear Regression and Correlation Between the Most Significant Metabolite (5-Methyl-2-Furoic Acid) and Diet Variables Among Controls

This supplementary material has been provided by the authors to give readers additional information about their work.

© 2019 Seow WJ et al. *JAMA Network Open.*

eTable 1. Spearman correlations between significant metabolites (<10% FDR) in controls only, cases only and overall study population

Metabolite 1	Metabolite 2	Controls		Cases		Overall	
		Correlation coefficient	P-value	Correlation coefficient	P-value	Correlation coefficient	P-value
pos_2.61_184.0325n	pos_2.61_127.0382m/z	0.89	9.98×10^{-230}	0.88	1.87×10^{-206}	0.89	$<9.98 \times 10^{-230}$
neg_2.60_369.0408m/z	pos_2.61_127.0382m/z	0.77	8.31×10^{-95}	0.68	5.93×10^{-52}	0.74	6.18×10^{-150}
pos_2.61_184.0325n	neg_2.60_369.0408m/z	0.69	3.04×10^{-57}	0.62	3.54×10^{-38}	0.67	9.02×10^{-101}

Abbreviations: FDR, false discovery rate

© 2019 Seow WJ et al. *JAMA Network Open.*

eTable 2. Associations between urinary metabolites and lung cancer risk, stratified by lung cancer subtypes

Lung cancer subtype	Metabolite[a]	Cases	Controls	OR (95% CI)[b]	P-value
Adenocarcinoma[c]	pos_2.61_127.0382mz	135	289	0.57 (0.43-0.76)	0.00012
	neg_2.60_369.0408mz	135	289	0.97 (0.96-0.98)	0.00013
	pos_2.61_184.0325n	135	289	0.53 (0.39-0.74)	0.00011
Non-adenocarcinoma[d]	pos_2.61_127.0382mz	28	289	0.51 (0.29-0.89)	0.017
	neg_2.60_369.0408mz	28	289	0.96 (0.93-0.99)	0.0035
	pos_2.61_184.0325n	28	289	0.44 (0.23-0.83)	0.012
Unclassified	pos_2.61_127.0382mz	104	289	0.58 (0.42-0.79)	0.00072
	neg_2.60_369.0408mz	104	289	0.97 (0.95-0.99)	0.00079
	pos_2.61_184.0325n	104	289	0.58 (0.41-0.82)	0.0024

Abbreviations: OR, odds ratio; CI, confidence interval

[a]Metabolites were natural log transformed.

[b]All models were adjusted for age, BMI, history of respiratory diseases and passive smoking.

[c]Adenocarcinoma cases include (8140/3) Adenocarcinoma, (8250/3) Bronchiolo-alveolar adenocarcinoma, (8251/3) Alveolar adenocarcinoma, (8260/3) Papillary adenocarcinoma and (8480/3) Mucinous adenocarcinoma.

[d]Non-adenocarcinoma cases include: carcinoma (2), small cell carcinoma (1), squamous cell carcinoma (9), neuroendocrine carcinoma (1), mucoepidermoid carcinoma (1) and adenosquamous carcinoma (14).

© 2019 Seow WJ et al. *JAMA Network Open.*

eTable 3. Associations between the most significant metabolite (5-methyl-2-furoic acid) and lung cancer in tertiles, stratified by lung cancer subtypes

Lung cancer subtype	Metabolite tertile	Cases	Controls	OR (95% CI)[a]	P-value	P-trend
Adenocarcinoma[b]	1st Tertile	70	96	1.00	-	0.0023
	2nd Tertile	35	95	0.55 (0.33-0.91)	0.021	
	3rd Tertile	30	96	0.45 (0.26-0.77)	0.0035	
Non-adenocarcinoma[c]	1st Tertile	15	96	1.00	-	0.15
	2nd Tertile	6	95	0.42 (0.15-1.19)	0.10	
	3rd Tertile	7	96	0.52 (0.20-1.37)	0.19	
Unclassified	1st Tertile	51	96	1.00	-	0.0043
	2nd Tertile	27	95	0.48 (0.27-0.86)	0.014	
	3rd Tertile	25	96	0.44 (0.25-0.79)	0.0063	

Abbreviations: OR, odds ratio; CI, confidence interval

[a]All models were adjusted for age, BMI, history of respiratory diseases and passive smoking.

[b]Adenocarcinoma cases (new) include (8140/3) Adenocarcinoma, (8250/3) Bronchiolo-alveolar adenocarcinoma, (8251/3) Alveolar adenocarcinoma, (8260/3) Papillary adenocarcinoma and (8480/3) Mucinous adenocarcinoma.

[d]Non-adenocarcinoma cases include: carcinoma (2), small cell carcinoma (1), squamous cell carcinoma (9), neuroendocrine carcinoma (1), mucoepidermoid carcinoma (1) and adenosquamous carcinoma (14).

© 2019 Seow WJ et al. *JAMA Network Open.*

eTable 4. Identification of the top three metabolites

Metabolite	Feature ID	Method	Ion	m/z	Retention time (min)	Fragments
5-methyl furan-2-carboxylic acid<br>(5-methyl-2-furoic acid)<br>HMDB #:<br>HMDB59735	2.61_127.0382m/z[a]	RP UPLC-MS/MS (ESI+)	$[M+H^+]^+$	127.038	2.621	71, 81, 109, 113
		RP UPLC-MS/MS (ESI-)	$[M-H^+]^-$	125.02	2.713	69, 79, 81, 97, 107
UA	2.61_184.0325n	RP UPLC-MS (ESI+)	$[M+H^+]^+$	185.025	2.607	The intensity of this ion is too low to obtain fragmentation information
UA	2.60_369.0408 m/z	RP UPLC-MS (ESI-)	$[M+FA-H^+]^-$	369.040	2.60	The ion $[M+H^+]^+$<br>325.055 and $[M+FA-H^+]^-$<br>are too low intensity to obtain fragmentation information

Abbreviation: UA, unannotated; ESI - electrospray ionization

[a]The fragmentation pattern of this feature has been identified to a level of assignment 2 (putatively annotated compound) and is matched to the fragmentation of the metabolite available on the online database METLIN. RP UPLC-MS – reverse phase ultrahigh performance mass spectrometry.

© 2019 Seow WJ et al. *JAMA Network Open.*

eTable 5. Associations between neg_2.60_369.0408mz, pos_2.61_184.0325n and lung cancer, by tertiles

Log Metabolite tertiles	Lung cancer	Non-lung cancer	OR (95% CI)[a]	P-value	P-trend
neg_2.60_369.0408mz					
1st Tertile (median = log0[b])	153	104	1.00	-	1.17×10^{-5}
2nd Tertile (median = 6.9)	62	87	0.47 (0.31-0.73)	0.00061	
3rd Tertile (median = 8.9)	60	98	0.40 (0.26-0.62)	3.18×10^{-5}	
pos_2.61_184.0325n					
1st Tertile (median = 9.2)	129	95	1.00	-	
2nd Tertile (median = 9.8)	75	94	0.58 (0.38-0.88)	0.011	0.00061
3rd Tertile (median = 10.5)	64	98	0.48 (0.31-0.74)	0.00089	

Abbreviations: OR, odds ratio; CI, confidence interval

[a]All models were adjusted for age, BMI, history of respiratory diseases and passive smoking.

[b]This group is composed of zero values.

© 2019 Seow WJ et al. *JAMA Network Open.*

eTable 6. Univariate linear regression and correlation between the most significant metabolite (5-methyl-2-furoic acid) and diet variables among controls

Diet variable	Regression coefficient	P-value[a]	Spearman correlation coefficient	P-value[b]
Egg	0.0069	0.0059	0.13	0.030
Soy food[a]	0.0010	0.0072	0.21	0.00041
Fish	0.0025	0.070	0.10	0.089
Crucifers	0.0011	0.11	0.089	0.13
All vegetables	0.0004	0.13	0.10	0.092
Meat	0.0017	0.21	0.071	0.23
Allium	-0.0067	0.24	0.045	0.45
Poultry	0.0028	0.29	0.014	0.82
Watermelon	0.0004	0.47	0.00093	0.99
Fruit	-0.0001	0.63	0.018	0.76
Rice	-0.0003	0.64	-0.069	0.24

Abbreviations: FDR, false discovery rate
[a]All models were adjusted for age, BMI, history of respiratory diseases and passive smoking. [b]Dietary soy food intake includes soy milk, tofu, fried tofu, dried or pressed tofu, fresh green soy beans, dry soy beans, soy sprouts, and other soy products.

© 2019 Seow WJ et al. *JAMA Network Open.*
